# A Pilot Study of a Pictorial Bilingual Nutrition Education Game to Improve the Consumption of Healthful Foods in a Head Start Population

**DOI:** 10.3390/ijerph9041319

**Published:** 2012-04-16

**Authors:** Veronica Piziak

**Affiliations:** Division of Endocrinology, Scott & White Healthcare, The Texas A&M Health Science Center College of Medicine, 2401 South 31st Street, Temple, TX 76508, USA; Email: vpiziak@swmail.sw.org; Tel.: +1-254-215-0316; Fax: +1-254-215-0325

**Keywords:** preschool children, nutritional intervention

## Abstract

The prevalence of early childhood obesity has increased dramatically particularly among the Mexican American population. Obesity leads to earlier onset of related diseases such as type 2 diabetes. The Head Start population of Texas is largely Mexican American. Dietary intake in this population demonstrated a diet very low in fiber, high in salt, and containing excessive calories with a low intake of fruit and vegetables. This study was performed in a Texas Head Start population to evaluate a bilingual pictorial nutrition education game. Acceptance of the bilingual concept and the game had been previously studied in a Head Start population in five Texas counties. The effectiveness in producing a change in eating habits was studied as a pilot project 413 children and their parents at the Bastrop County Head Start. Parents were asked to supply data about at home food frequency at the beginning and the end of the school year and the results compared. The parents were given a demonstration of the educational objectives and the students played the game throughout the year. By the end of the school year there was a statistically significant increase in the vegetables offered to this population both during the week at home (*p* = 0.009) and on the weekends (*p* = 0.02).

## 1. Introduction

The prevalence of overweight and obesity among preschool children has been increasing world wide for over 40 years [1,2,3]. Mexican-American children have a higher prevalence than Caucasian children [4]. Preschool children from low income families are particularly affected with 1 in 7 children noted to be obese in 2009 [5]. The health consequences of obesity such as diabetes, previously limited to adults, are seen in younger age groups [6]. Obesity and ethnicity in Mexican American children markedly increases the risk of early onset Type 2 diabetes and early metabolic syndrome. Recent evidence suggests that obese children also have lower bone mineral density [7].

Many factors lead to increased weight in high risk preschool children but recent evidence suggests that dietary changes particularly increased consumption of sugar sweetened beverages [8], decreased consumption of high fiber foods such as vegetables and increased fat consumption are implicated [9]. Increased screen time as TV watching and computer time leads to decreased exercise. In addition, TV advertising on programming targeted toward children is heavily weighted toward food that is not nutritious and has been shown to influence food choices by children [10]. Targeting nutritional education in preschool children is essential to establish long term eating habits, but the challenges are substantial. The Head Start setting is an important opportunity for nutrition educational activities that are participatory and culturally relevant. This population is also at high risk for obesity because of ethnic and economic composition. 

In the border state of Texas the poverty level is high, particularly in the Mexican-American population, and many of the families qualify for Head Start. The prevalence of obesity in the Head Start population surveyed is 20%, far above the national average [11]. In the Head Start Centers of Texas, many children speak Spanish or are bilingual, and reading skills are low at this age. Limited funding can also be a problem. In addition, access to dietary education for families is suboptimal. Studies of dietary habits of the population showed excess calories, fat, concentrated carbohydrates and sodium were taken and insufficient amounts of fiber, potassium, and vitamin A were eaten [12]. 

The purpose of this study was to test the effectiveness of a bilingual nutrition game to increase the servings of healthful foods particularly vegetables, fruit, and water offered to children and decrease the servings of sugar sweetened beverages in the Head Start population.

## 2. Methods

### 2.1. Description of Game

**Figure 1:**
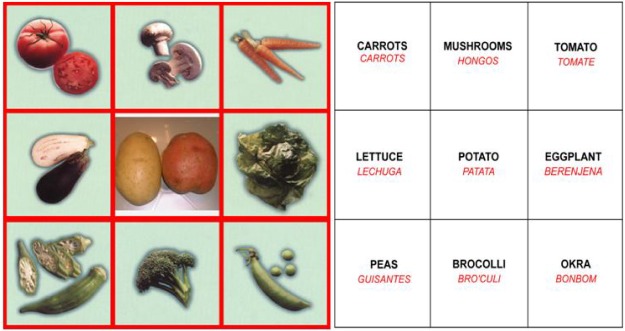
Game board: Front (pictures), Back (words).

**Figure 2:**
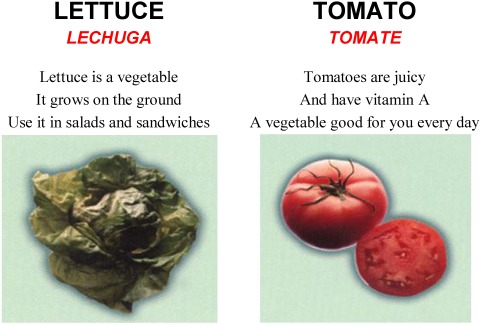
Game cards: pictures and words.

The nutrition education game is patterned after Loteria a popular pictorial bingo game in the Mexican-American community. The moderator of the game shows a card with a picture and tells a story or recites a rhyme to describe the picture. The players then put a token on their game boards if they have the picture. This game lends itself to nutrition education. The cards and boards show color images of culturally appropriate food and the reverse side gives the names in English and Spanish which may be used to improve reading skills (Figure 1). Locally popular ethnic foods were included as suggested by the teachers in focus groups. All of the fruits and vegetables in the game are available in the area and popular ethnic foods appear more often on the game boards. The cards contain the name of the food in English and Spanish and a set of rhymes with information in English or Spanish about the foods (Figure 2). There are several ways to use the game in the Head Start setting: the deck may be used as a set of flash cards to acquaint the children with different types of food and their place in a healthy diet; both the game boards and the deck may be used to play a variety of types of nutritional bingo [13]. The teachers were allowed to play the game in either English or Spanish. They could adapt the length of game play for their schedule and attention span of the particular class. The rhymes from the cards could be recited when the children were eating their meals and snacks since many of the foods featured in the game were served by Head Start. Game modifications were made on the basis of suggestions with the teachers at the pilot site in central Texas.

### 2.2. Study Population

The project was approved by the Scott and White IRB. The game was provided to Head Start centers in three Texas border counties and two central Texas counties for use with approximately 10,000 children. Head Start is a government funded locally operated preschool program providing basic education for children of low income families. Most of the children are from 3–5 years of age. The pilot study examines the results in 413 children in one central Texas county where the Head Start population is 57.3% Mexican-American and 56.8% have a family history of diabetes. The parents were acquainted with the game in a meeting where parents were encouraged to play for prizes and counseled about healthy food choices. At the beginning and end of the school year parents completed a questionnaire about the food offered to the children at home on Monday thru Friday and on the weekends. The parents were asked to document the number of times a day that the children were served milk and soda and water, fruit and vegetables during the week and on weekends. They were not asked to estimate the amount consumed. The parents received instructions about how to complete the questionnaire. The parents were asked to document milk and soda and water, fruit and vegetable served. Head Start provides a healthy diet and structured exercise during the school day on Monday thru Friday. The teachers were instructed about the game and given basic nutrition counseling during a training session before the start of the school year. The game was played by the children at least twice a week and the children were encouraged to recite the rhymes that were on the cards as they were presented.

### 2.3. Data Analysis

To preserve the privacy of the children anonymous aggregate data from the entire county was analyzed. The signed rank test was used to test statistical significance of the difference from the beginning of the school year to the end of the year for the water, milk, soda, fruit, and vegetable served.

## 3. Results and Discussion

Results are shown in Table 1. The mean value for any given parameter was the mean servings of a given food per day for the entire population for the specified time period (Monday – Friday outside of Head Start or on weekends). The difference in the “means” was the difference between the mean value before and after the game was used. Before the intervention 38 parents reported no vegetable served on weekends and 16 reported no vegetable served at all. At the end of the study only 12 families reported no vegetable served on weekends and only 4 reported no vegetable served at any time. There was a statistically significant increase in vegetable served outside of Head Start both after school during the week and on the weekends. There was no change in the amount of exercise in the group over the school year. The study was unable to demonstrate any other statistically significant changes in dietary habits. However, feedback from a focus group of teachers demonstrated that the game was important in promoting recognition of a variety of foods by the children. The repetition of the rhymes could be used to promote the importance of increased water intake and reinforce the desirable decrease in drinking of sugar sweetened beverages and the routine intake of foods with high sugar content which is the policy of Head Start. The teachers felt that they would like to continue to use the game.

**Table 1:** Mean Values for Daily Servings of Foods and Water by the Study Population Before and after the Intervention.

***Servings per day(Mean for the population)***	***Before***	***After***	***Difference***
**Water on Saturday and Sunday**	3.99	3.97	**–0.02**
**Milk on Saturday and Sunday**	3.047	3.046	**–0.021**
**Soda on Saturday and Sunday**	1.78	1.62	**–0.16**
**Fruit on Saturday and Sunday**	3.03	3.04	**0.01**
**Vegetables on Saturday and Sunday**	2.34	2.68	0.34 *
**Water on Monday through Friday**	3.98	4.07	**0.09**
**Milk on Monday through Friday**	3.34	3.26	**–0.08**
**Soda on Monday through Friday**	1.64	1.57	**–0.07**
**Fruit on Monday through Friday**	3.32	3.28	**–0.04**
**Vegetables on Monday through Friday**	**2.57**	**2.94**	0.37 **

* *P* value = 0.02; ** *P* value = 0.009.

Recently, multiple initiatives have been directed toward changing the eating and exercise habits of the preschool population to attempt to decrease obesity and thus prevent the early onset of hypertension, diabetes and metabolic syndrome [14]. A structured education program for preschool children and parents has resulted in an increase in fruit and vegetable consumption [15]. Head Start particularly has made excellent progress in instituting education programs for parents and children [16]. The findings from this pilot study in cooperation with a Texas county Head Start show that vegetable servings may also be improved in the age group by using a simple pictorial nutrition education game that may be played in either English or Spanish and incorporates elements of the Mexican American culture familiar to the majority of the students and their parents. This familiarity simplifies instruction of the teachers and parents. The game is also inexpensive to reproduce helping to control costs. This study only encompasses one of the 5 counties and the data from the total population is still being analyzed. In the future the parents will be asked to play the game with the children and incentives will be provided to families who participate. An exercise initiative using cartoon characters leading the children in age appropriate exercise routines and counseling about healthy foods has been tested and accepted by the teachers and will be studied in the pilot county as well.

## References

[1] Ogden C.L., Troiano R.P., Briefel R.R., Kuczmarski R.J., Flegal K.M., Johnson C.L. (1997). Prevalence of overweight among preschool children in the United States, 1971 through 1994. Pediatrics.

[2] Ogden C.L., Flegal K.M., Carroll M.D., Johnson C.L. (2002). Prevalence and trends in overweight among US children and adolescents, 1999–2000. JAMA.

[3] de Onis M., Blössner M., Borghi E. (2010). Global prevalence and trends of overweight and obesity among preschool children. Am. J. Clin. Nutr..

[4] Ogden C.L., Carroll M.D., Flegal K.M. (2008). High body mass index for age among US children and adolescents, 2003–2006. JAMA.

[5] (2009). Pediatric Nutrition Surveilance System. www.cdc.gov/obesity/childhood/data.html.

[6] Haines L., Wan K.C., Lynn R., Barrett T.G., Shield J.P. (2007). Rising incidence of type 2 diabetes in children in the UK. Diabetes Care.

[7] Mughal M.Z., Khadilkar A.V. (2011). The accrual of bone mass during childhood and puberty. Curr. Opin. Endocrinol. Diabetes Obes..

[8] Nelson J.A., Carpenter K., Chiasson M.A. (2006). Diet, activity, and overweight among preschool-age children enrolled in the special supplemental nutrition program for Women, Infants, and Children (WIC). Prev. Chronic Dis..

[9] Barquera S., Campirano F., Bonvecchio A., Hernández-Barrera L., Rivera J.A., Popkin B.M. (2010). Caloric beverage consumption patterns in Mexican children. Nutr. J..

[10] Dixon H.G., Scully M.L., Wakefield M.A., White V.M., Crawford D.A. (2007). The effects of television advertisements for junk food *versus* nutritious food on children’s food attitudes and preferences. Soc. Sci. Med..

[11] Piziak V.K., Morgan-Cox M.A., Tubbs J., Hasan M. (2010). Elevated body mass index in Texas Head Start children: A result of heredity and economics. South. Med. J..

[12] Mier N., Piziak V., Kjar D., Castillo-Ruiz O., Velazquez G., Alfaro M.E., Ramirez J.A. (2007). Nutrition provided to Mexican-American preschool children on the Texas-Mexico border. J. Am. Diet. Assoc..

[13] Mier N., Piziak V., Valdez L. (2005). Ultimate nutrition game for Mexican-American preschoolers. J. Nutr. Educ. Behav..

[14] Hesketh K.D., Campbell K.J. (2010). Interventions to prevent obesity in 0–5 year olds: An updated systematic review of the literature. Obesity (Silver Spring).

[15] Witt K., Dunn K. (2011). Increasing fruit and vegetable consumption among preschoolers: Evaluation of color me healthy. J. Nutr. Educ. Behav..

[16] Larson N., Ward D.S., Neelon S.B., Story M. (2011). What role can child care settings play in obesity prevention? A review of the evidence and a call for research efforts. J. Am. Diet. Assoc..

